# Cytochrome P450 *BsCYP99A44* and *BsCYP704A177* Confer Metabolic Resistance to ALS Herbicides in *Beckmannia syzigachne*

**DOI:** 10.3390/ijms232012175

**Published:** 2022-10-12

**Authors:** Shuang Bai, Mengjie Yin, Qinghao Lyu, Bo Jiang, Lingxu Li

**Affiliations:** College of Plant Health and Medicine, Qingdao Agricultural University, Qingdao 266109, China

**Keywords:** *Beckmannia syzigachne*, cytochrome P450s, mesosulfuron-methyl, non-target-site resistance, mechanism

## Abstract

*Beckmannia syzigachne* is a noxious grassy weed that infests wheat fields in China. Previously, we identified that mesosulfuron-methyl resistance in a *B. syzigachne* population (R, SD04) was conferred by non-target resistance, such as cytochrome P450 mixed-function oxidases (P450s)-based metabolism. RNA sequencing and real-time PCR (qRT-PCR) were used to discover potential P450s-resistant-related genes. Five cytochrome P450s (*CYP704A177*, *CYP96B84*, *CYP71D7*, *CYP93A1,* and *CYP99A44*) were found to be highly expressed in R plants. In this study, *CYP99A44* and *CYP704A177* were cloned from *B. syzigachne* and transferred into *Arabidopsis thaliana* to test the sensitivity of *Arabidopsis* with and without P450s genes to mesosulfuron-methyl and other acetolactate synthase (ALS)-inhibiting herbicides. Transgenic *Arabidopsis* overexpressing *CYP99A44* became resistant to the sulfonylurea herbicide mesosulfuron-methyl, but showed no resistance to pyroxsulam, imazethapyr, flucarbazone, and bispyribac-sodium. Notably, those overexpressing *CYP704A177* showed resistance to pyroxsulam and bispyribac-sodium, but not to mesosulfuron-methyl, imazethapyr, and flucarbazone. These results indicated that *B. syzigachne* and transgenic *Arabidopsis* displayed different cross-resistance patterns to ALS-inhibiting herbicides. Subcellular localization revealed that *CYP99A44* and *CYP704A177* protein were located in the endoplasmic reticulum. Furthermore, these results clearly indicated that *CYP99A44*-mediated mesosulfuron-methyl resistance in *B. syzigachne* and *CYP704A177* may be involved in *B. syzigachne* cross-resistance to pyroxsulam and bispyribac-sodium.

## 1. Introduction

In agricultural production, herbicides have become effective tools for weed control; however, owing to the selective pressure of herbicides, weeds have evolved resistances worldwide, resulting in a substantial threat to global agriculture [1,2,3]. Weed resistance mechanisms are usually divided into two categories: namely, target-site (TSR) and non-target-site resistance (NTSR) [4]. Target-site resistance can occur due to amino acid substitutions resulting in a lower binding affinity for herbicides at the target enzyme [5]. Overproduction of target-site proteins resulting from increased expression or gene duplication is another mechanism for target-site resistance [6,7]. The molecular mechanism of target-site resistance is relatively easy to analyze because the target enzymes of most herbicides is known [4]. Nonetheless, there is minimal information on the molecular mechanisms of herbicide resistance at non-target-sites, although it poses a greater threat to weed control, and can confer resistance to multiple herbicides within the same or even different herbicide modes-of-action.

Most non-target-site resistant mechanisms are associated with enhanced herbicide metabolism (hereinafter referred to as metabolic resistance) and impaired translocation [5,8,9]. A main culprit of metabolic resistance is plant cytochrome P450 monooxygenases (thereafter referred to as P450s) [4,5,10], which form a large family that catalyze a wide variety of monooxygenation/hydroxylation reactions [11] and participates in various biochemical pathways to produce primary and secondary metabolites [12]. A handful of P450s in crops and grass weeds have been identified to deliver metabolic resistance to herbicides with different modes of action [13]. For example, genetic mapping and complementation tests revealed that the P450 gene *CYP81A6* is involved in bensulfuron-methyl tolerance in rice [14]. CYP81As in *Echinochloa phyllopogon* have also been shown to metabolize multiple herbicides [3,15,16]; however, only a few genes have been found to contribute to NTSR in weeds. Currently, our understanding of metabolic herbicide resistance in weed species is limited due to the complexity of metabolic resistance and the diversity of plant P450s [17].

American sloughgrass (*Beckmannia syzigachne*) is an annual grass weed and is often a problematic weed in wheat fields within a rice-wheat rotation [18], and mesosulfuron-methyl, an acetolactate synthase (ALS) inhibiting herbicide, is commonly used for control. To date, many studies showed that the continuous application of mesosulfuron-methyl to control *B. syzigachne* has led to the serious resistance problem [19,20]. Most resistance in *B. syzigachne* to mesosulfuron-methyl is due to target-site resistance [18,21], but cases of non-target-site resistance mechanisms exist and have not been clearly articulated. In our previous study, a purified *B. syzigachne* biotype (R) without any known resistant mutations in the ALS gene showed 4.1-fold resistance to mesosulfuron-methyl based on GR_50_ values and showed cross-resistant to pyroxsulam, imazethapyr, flucarbazone, and bispyribac-sodium. Further experimentation found the R biotype degraded more mesosulfuron-methyl than the susceptible biotype (S) [20]. Some cytochrome P450 genes such as *CYP704A177, CYP96B84, CYP71D7, CYP93A1,* and *CYP99A44* may be involved in the resistance of *B. syzigachne* according to RNA-Seq analyses. Moreover, the known cytochrome P450 inhibitor, malathion, was found to reverse this resistance; therefore, it is likely that P450s are involved in mesosulfuron-methyl resistance in R plants [20]. In this study, based on the transcriptome sequencing results, we report the identification and functional characterization of the P450 gene *CYP99A44* (GenBank accession number OP243711), which plays a significant part in mesosulfuron-methyl resistance in *B. syzigachne*. We also demonstrated that the overexpression of *CYP704A177* (OP243712) confers *B. syzigachne* resistance to other ALS-inhibiting herbicides, including pyroxsulam and bispyribac-sodium.

## 2. Results

### 2.1. RNA-Seq and Bioinformatics Analysis

Prior work showed P450s are involved in mesosulfuron-methyl resistance in the R *B syzigachne* population and here we used RNA-Seq to identify the responsible genes. In this study, averages of 29,743,334 clean reads and 445,504,726 clean bases were obtained per sample (Supplementary Table S1). Furthermore, we obtained 53,651 unigenes with a mean length of 964.98 bp, and their shortest and longest sequences measured 201 and 15,352 bp, respectively (Supplementary Table S2).

**Figure 1:**
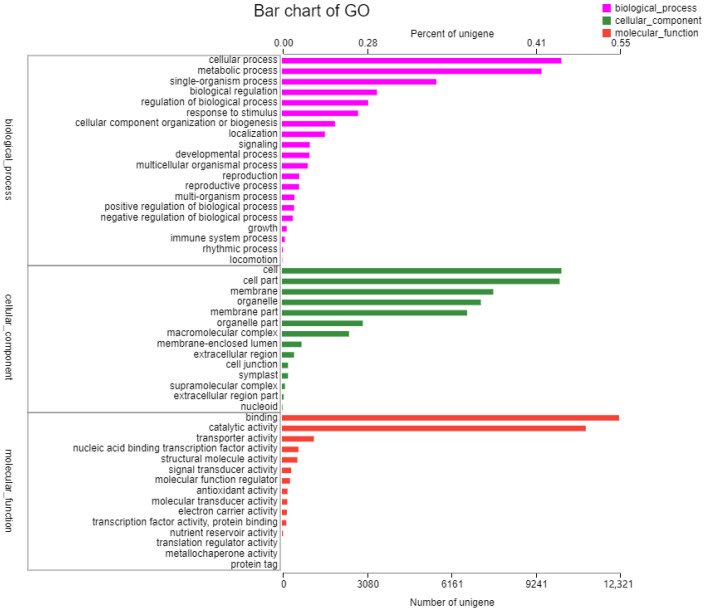
Gene Ontology (GO) function classification of the annotated unigenes in resistant *Beckmannia syzigachne*. The unigenes were allocated to three categories: biological process, cellular component, and molecular function. The unigenes sequence of resistant (R) *B*. *syzigachne* have been deposited in the NCBI Sequence Read Archive (SRA) database with accession number SRR21047671.

**Figure 2:**
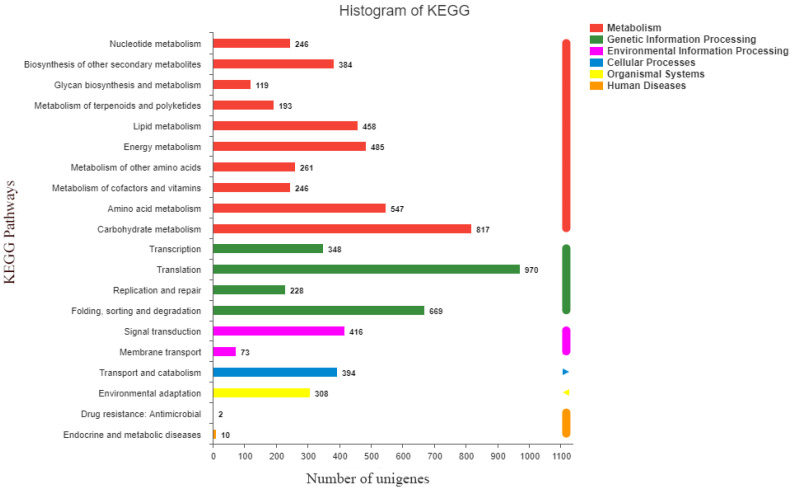
Kyoto Encyclopedia of Genes and Genomes (KEGG) function classification results of the annotated unigenes in resistant *Beckmannia syzigachne*. The x and y axes indicate KEGG pathways and the number of genes, respectively.

The sequences were annotated using the Gene Ontology (GO), Clusters of Orthologous Groups of proteins (COG), Kyoto Encyclopedia of Genes and Genomes (KEGG), NR, Swiss-Prot, and Pfam databases. The GO database assigned 22,167 unigenes to 19 functional regions, and the top three GO annotation were cellular process (33.78%), metabolic process (29.50%), and biological regulation (10.32%) (Figure 1). Moreover, 52,270 unigenes were categorized into 23 COG classifications, and 7088 assembled sequences were mapped to the reference canonical pathway in the KEGG database. According to the KEGG metabolic pathway, there were 817 genes involved in carbohydrate metabolism and 416 genes involved in signal transduction in environmental information processing. Furthermore, 2215 genes were involved in genetic information processing and 308 genes were related to environmental adaptation (Figure 2). The NR, Swiss-Prot and Pfam databases annotated 23,764, 17,271, and 21,506 genes, respectively.

### 2.2. Differential Expression Analysis and Validation of P450s

Differential expression analysis of RNA-Seq data between the TR (R population at 24 h after mesosulfuron-methyl treatment) and CK (R population without mesosulfuron-methyl treatment) groups found a total of 4865 genes, in which 2681 genes were up-regulated and 2184 genes were down-regulated in the TR (Supplementary Figure S1).

P450s, GSTs, GTs, and ABC transporter proteins were selected as key enzymes related to metabolism or signal transduction according to previous studies [9,22]. Of these, 89 genes were up-regulated in *B. syzigachne* plants after mesosulfuron-methyl treatment, including 47 P450s, 28 GSTs, 3 GTs, and 11 ABC transporters (Supplementary Tables S3–S6). Our previous work indicated that P450 were the likely player in metabolic resistance and thus focused on P450s.

To confirm RNA-Seq data, 29 P450s differentially expressed genes (DEGs) by increasing selection criteria were selected for further verification using the real time PCR (qRT-PCR). Expression differences were compared between the TR and CK group after mesosulfuron-methyl treatment at 12, 24, and 48 h (Table 1). Out of the 29 genes, we observed that four cytochromes P450 DEGs were up-regulated consistently in *B. syzigachne* at 12, 24, and 48 h after mesosulfuron-methyl treatment, including *CYP704C1* (TRINITY_DN11725_c0_g1), which has been named *CYP704A177*; *CYP96B84* (TRINITY_DN11183_c0_g1); *CYP71D7* (TRINITY_DN13492_c1_g1); and *CYP93A1* (TRINITY_DN13901_c6_g2). In addition, *CYP99A44* showed relative high expression in omics analysis and 48 h after mesosulfuron-methyl treatment by qRT-PCR. It was identified as a fenoxaprop-*p*-ethyl metabolism related gene in our previous study (annotated as *CYP99A2*) [23]. Therefore, these genes including *CYP99A44* were considered the major genes involved in mesosulfuron-methyl resistance in *B. syzigachne.*

### 2.3. Full Sequence Cloning and Analysis of CYP99A44 and CYP704A177

**Figure 3:**
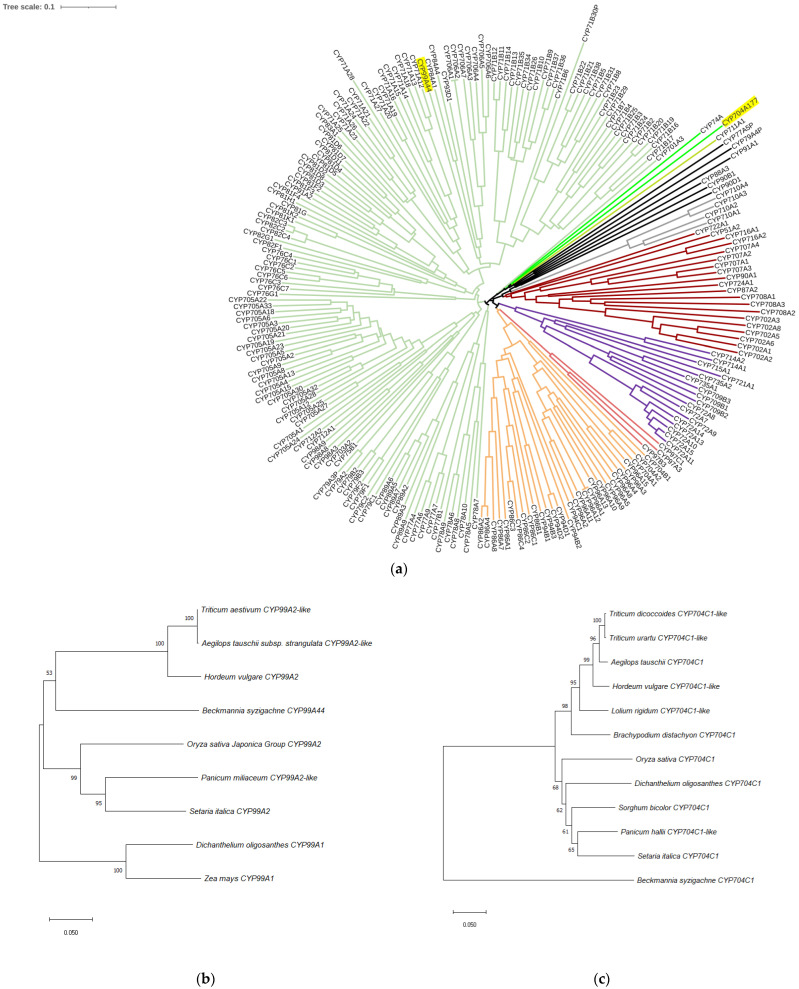
Sequence analysis of resistant *Beckmannia syzigachne* P450 proteins. (**a**) Multiple sequence alignment of the *B. syzigachne CYP99A44* and *CYP704A177* (highlighted in yellow background) and the 227 P450s from *Arabidopsis* species. P450 groups thought to comprise individual clades are colored. Nearest-neighbor analyses of (**b**) *CYP99A44* and (**c**) *CYP704A177*.

Next, the full-length coding sequences of the *CYP99A44* and *CYP704A177* genes were amplified from *B. syzigachne*. Gene conserved domains were analyzed using the National Center for Biotechnology Information conserved domain tool (http://www.ncbi.nlm.nih.gov/structure/cdd/wrpsb.cgi (accessed on 9 October 2022)), confirming that these two genes belong to the P450 superfamily (Supplementary Figure S2). Sequence analysis of these *B. syzigachne* genes and 227 P450 *Arabidopsis* genes showed that *CYP99A44* clustered in the CYP71 clan, and *CYP704A177* clustered in the CYP704 family of CYP74 clan [11] (Figure 3a). Nearest-neighbor analyses of the *CYP99A44* sequence indicated that *B. syzigachne CYP99A44* has close evolutionary relationship with *Hordeum vulgare CYP99A2*, *Aegilops tauschii CYP99A2*-like, and *Triticum aestivum CYP99A2*-like (Figure 3b). *CYP704A177* had a relatively close evolutionary relationship with *Brachypodium distachyon CYP704C1* (Figure 3c).

### 2.4. Sensitivity to Mesosulfuron-Methyl and Other Herbicides of Arabidopsis Overexpressing CYP99A44 and CYP704A177

**Figure 4:**
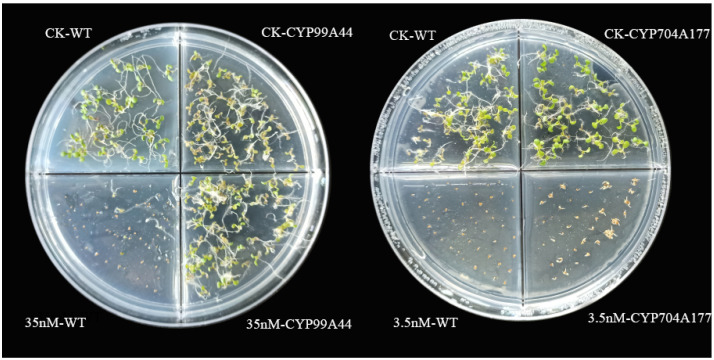
Susceptibility of in *Arabidopsis* transformed with *CYP99A44* or *CYP704A177* to mesosulfuron-methyl. Seedlings of the transgenic *Arabidopsis* grown on media containing no herbicide (CK), at 3.5 nM (**right**) and 35 nM (**left**) mesosulfuron-methyl.

Full-length coding sequences of *B. syzigachne CYP99A44* and *CYP704A177* were introduced into *Arabidopsis thaliana* (ecotype Columbia-0), under the control of the cauliflower mosaic virus (CaMV) 35S promoter. The independent T3 transformant lines were placed in Murashige and Skoog medium containing mesosulfuron-methyl herbicide, and the *Arabidopsis* seeds overexpressing *CYP99A44* could grow normally at 35 nM while the wild type (WT) was completely killed (Figure 4), indicating that *CYP99A44* endowed *Arabidopsis* with mesosulfuron-methyl resistance; however, the growth of the *CYP704A177* transformants was inhibited at 3.5 nM (Figure 4). This result suggests that *CYP704A177* may not be a major player in the metabolism of mesosulfuron-methyl in *B. syzigachne.*

**Figure 5:**
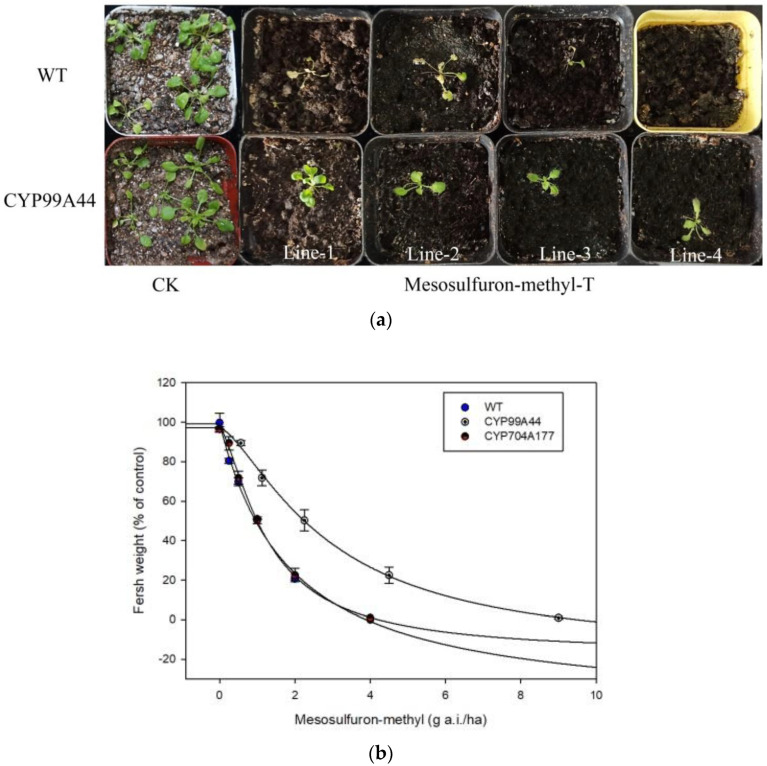
(**a**) Growth response of *Arabidopsis* (WT) and those transformed with *CYP99A44* to mesosulfuron-methyl (Lines 1–4). Growth status was checked at 14 d after treatment at the rate of 0 (CK) or 3.38 g a.i./ha (Mesosulfuron-methyl-T). (**b**) Dose–response curves of independent transgenic lines to mesosulfuron-methyl. Bars represent SE (*n* = 3).

To verify the sensitivity of the *Arabidopsis* lines overexpressing *CYP99A44* or *CYP704A177* to mesosulfuron-methyl, whole-plant dose–response experiments were conducted. After 14 d of treatment (DAT), the plants were cut to determine above-ground biomass. Herbicide concentration resulting in 50% growth reduction in fresh weight (GR_50_ value) of *CYP99A44* was 2.88 g a.i./ha, which was 2.25-fold more resistant to mesosulfuron-methyl than the WT (Table 2). The GR_50_ of *CYP704A177* was 1.83 g a.i./ha with a resistance index of 1.43 to the WT (Table 2, Figure 5b). This was consistent with the phenotype obtained in the above-mentioned Petri dish (Figure 5a), suggesting that *CYP99A44* but not *CYP704A177* endowed *Arabidopsis* mesosulfuron-methyl resistance.

**Figure 6:**
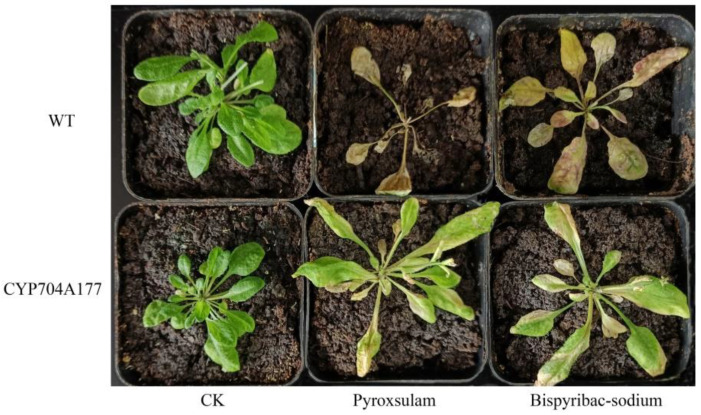
Growth response of *Arabidopsis* transformed with *CYP704A177* to pyroxsulam (2.81 g a.i./ha) and bispyribac-sodium (7.5 g a.i./ha).

To check for cross resistance, overexpression lines were sprayed with pyroxsulam, imazethapyr, flucarbazone, and bispyribac-sodium at 1/4 of recommended label rates. No significant resistance of *Arabidopsis* lines overexpressing *CYP99A44* was identified to those tested ALS inhibitors. Notably, plants overexpressing *CYP704A177* in comparison to the WT were more resistant to pyroxsulam and bispyribac-sodium at 2.81 and 7.5 g a.i./ha (1/4 of the recommended dose in the field), respectively, than WT (Figure 6). Nevertheless, there were no survivors of *CYP704A177* at the tested concentrations of imazethapyr and flucarbazone.

### 2.5. Subcellular Location of CYP99A44 and CYP704A177

**Figure 7:**
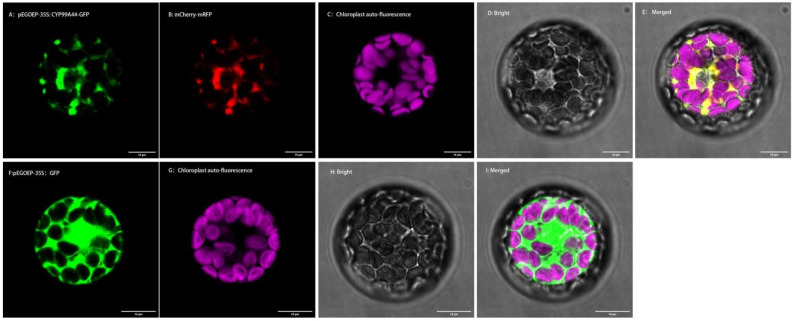
Subcellular localization of *CYP99A44*. (**A**): pEGOEP-35S: *CYP99A44*-GFP shown in green; (**B**): endoplasmic reticulum (ER) marker mCherry-mRFP shown in red; (**C**): chloroplast auto-fluorescence; (**D**): the bright-field; €: merged images; (**F**): pEGOEP-35S: GFP shown in green; (**G**): endoplasmic reticulum (ER) marker mCherry-mRFP shown in red; (**H**): the bright-field; (**I**): merged images. Scale bars: 10 μm in (**A**–**I**).

The *Arabidopsis* protoplast transit expression system was used to determine the subcellular localizations of *CYP99A44* and *CYP704A177*. Multiphoton confocal laser scanning microscopy of the transformed *Arabidopsis* protoplasts showed that the protoplast pEGOEP-35S: GFP alone had strong fluorescent signals in the cytoplasm, cell membrane, and nucleus (Figure 7F). When expressed together with *CYP99A44*, green and red fluorescent signals from pEGOEP-35S: *CYP99A44*-GFP and the endoplasmic reticulum marker mCherry-mRFP completely merged with the endoplasmic reticulum rendered in yellow (Figure 7E). This suggested that *CYP99A44* was localized to the endoplasmic reticulum. Furthermore, the subcellular location of *CYP704A177* showed the same result (Supplementary Figure S3).

## 3. Discussion

In our previous study, the *B. syzigachne* R population with NTSR exhibited resistance to mesosulfuron-methyl, pyroxsulam, imazethapyr, flucarbazone, and bispyribac-sodium. Moreover, P450s may be involved in mesosulfuron-methyl resistance in the R population [20]. Nevertheless, most of the available evidence for the involvement of P450s in herbicide resistance is indirect, that is, it is measured by P450 inhibitors, RNA-Seq, or transcript levels [22,24]. Although a large number of genes have been discovered in omics [25,26,27], few of them have been identified as being involved in specific herbicide metabolism [17,28,29]. The strongest evidence for P450 conferring herbicide resistance is the expression of the P450 allele in herbicide-sensitive individuals or surrogate organisms (such as *Arabidopsis*) and screening for reduced susceptibility [30,31]. From this, some P450 genes have been discovered. For example, *CYP81A10v7* endows *Lolium rigidum* with at least five modes of action across seven herbicide chemistries (e.g., ACCase-, ALS-, PSII-, HPPD- and tubulin-inhibiting herbicides) [28]. Variants of *CYP81A9* (Nsf1) have been reported to be responsible for the metabolism of nicosulfuron in sweet corn [32]. Higher-expression-level *CYP709C56* degrades mesosulfuron-methyl in *A. aequalis* [17], and *CYP77B34* from *Descurainia sophia* can confer *Arabidopsis* resistance to tribenuron-methyl, bromoxynil and pretilachlor [30]. Weeds are good materials for providing genetic resources for herbicide-resistant crops, and scientists have identified several CYP81A subfamily genes (*CYP81A12, CYP81A14, CYP81A15, CYP81A18, CYP81A21, CYP81A24,* and *CYP81A63*) that metabolize herbicides only in *E. phyllopogon* [3,15,16,33], and *CYP81A68* endows generalist metabolic resistance to penoxsulam and cyhalofop-butyl in *Echinochloa crus-galli* [34].

To reveal the P450 genes involved in mesosulfuron-methyl metabolism in R, in this study, *CYP704A177*, *CYP96B84*, *CYP71D7*, *CYP93A1,* and *CYP99A44* were screened by using RNA-Seq and qRT-PCR. This differed from the results that *CYP86B1* was involved in mesosulfuron-methyl resistance in other resistant *B. syzigachne* using RNA-Seq analysis [19]. This may depend on the intensity of herbicide or environmental selection, and because of the genetic diversity of plants, different P450 genes and other genes may be involved in different resistant weed species [5]. In this study, we characterized the functions of the candidate P450s and provided clear evidence that some P450 genes endowed *B. syzigachne* with herbicide resistance. Nevertheless, difficulties were encountered in obtaining the complete coding region of *CYP71D7, CYP96B84*, and *CYP93A1*. Therefore, this study focuses on the other two P450 genes. *CYP99A44* or *CYP704A177* genes were transferred into *Arabidopsis*, and their sensitivities to mesosulfuron-methyl and other ALS herbicides were observed. Our results indicated that *CYP99A44* exhibited resistance to mesosulfuron-methyl, but not to pyroxsulam, imazethapyr, flucarbazone, or bispyribac-sodium. Conversely, *CYP704A177* showed resistance to pyroxsulam and bispyribac-sodium, but not to mesosulfuron-methyl, imazethapyr, and flucarbazone at the tested doses. These results indicated that transgenic *Arabidopsis* and *B. syzigachne* displayed different cross-resistance to herbicides. These results were not surprising as plants possess hundreds of P450s with varying substrate specificities. *CYP99A44*-mediated *B. syzigachne* resistance to mesosulfuron-methyl and *CYP704A177* may be involved in *B. syzigachne* cross-resistance to pyroxsulam and bispyribac-sodium. This further verified the complexity of NTSR controlled by polygenic adaptation to herbicides. Meanwhile, *CYP99A44* and *CYP704A177* were localized in the endoplasmic reticulum, which accomplishes various types of molecular machines of proteins, including folding, signal transduction, quality control, and degradation [35]. This will lay a foundation for the study of gene-catalyzed metabolism of exogenous substances.

In this study, sequence analyses showed that *CYP99A44* and *CYP704A177* clustered in the CYP71 and CYP74 clan (Figure 3a). Previous phylogenetic analyses of plant P450s have shown that the CYP71 clan is the youngest clan [11,36], is highly proliferative, and includes P450s involved in the metabolism of most plant-specialized compounds [37]. In the CYP71 clan, members of distantly related subfamilies have been suggested to play a role in the oxidation of monoterpenoids and sesquiterpenoids [37]. The CYP76B and CYP71A families of the CYP71 clan have been reported to accept exogenous substrates and possess the capacity for heterologous degradation of xenobiotics [38,39], indicating considerable functional promiscuity. Therefore, the role of *CYP99A44* in the reduced sensitivity of transgenic *Arabidopsis* plants to mesosulfuron-methyl is not hard to understand. The CYP74 clan contains a limited number of subfamilies involved in generating signaling molecules, such as jasmonates, that metabolize oxygenated polyunsaturated C18 fatty acid hydroperoxides to oxylipids in the octadecanoid pathway, which is essential for host immunity and plant development [37,40]. Currently, there are few genes that have been shown to metabolize herbicides in the CYP74 clan; thus, further research is needed to determine how *CYP99A44* catalyzes mesosulfuron-methyl degradation. Zhao et al. [17] demonstrated that *CYP709C56* metabolizes mesosulfuron-methyl via O-demethylation in yeast expression experiments, and Iwakami et al. [33] reported that *CYP81A12* and *CYP81A21* similarly metabolize bensulfuron-methyl through O-demethylation. These examples provide a foundation for further research.

## 4. Materials and Methods

### 4.1. Plant Material Preparation and RNA-Seq

The ALS resistant *B. syzigachne* population (SD-04, referred to a R) used in this study was described in Wang et al. [20]. For all *B. syzigachne* experiments, plants were germinated as described in [23] and once seedlings had a 1 cm shoot length, 10 plants were transplanted into 12-cm diameter pots. In all *B. syzigachne* experiments, one plant per pot was considered one repetition. Pots were transferred to a greenhouse in Qingdao Agricultural University (temperature maintained at approximately 15 to 25 °C and natural sunlight) and watered as needed. For the RNA-Seq experiment, once plants reached the 3 to 4 leaf stage, they were sprayed with a herbicide emulsion using a compressed air, moving nozzle cabinet sprayer equipped with one Teejet 9503EVS flat fan nozzle and calibrated to deliver 450 L/ha at 0.28 MPa. The TR group was treated with technical grade mesosulfuron-methyl at a dosage of twice the recommended field dose of 30 g a.i./ha. Plants sprayed with an equal amount of dimethylbenzene emulsion served as control group (CK). The aboveground shoot tissue was harvested at 24 HAT and frozen in liquid nitrogen for RNA extraction. The experiment included three biological replicates of the CK and TR groups, with six samples prepared overall.

Total RNA was extracted using an EasyPure Plant RNA Kit (TransGen Biotech, Beijing, China); RNA quality was confirmed using 1% agarose gels, Qubit^®^ 3.0 Flurometer (Life Technologies, Carlsbad, CA, USA), and RNA Nano 6000 Assay Kit of the Bioanalyzer 2100 system (Agilent Technologies, Santa Clara, CA, USA). Three microgram of total RNA per sample was used to prepare the cDNA library using the method described by Chen et al. [41]. The 300-bp paired-end reads were generated using an Illumina Hiseq 150 platform.

Raw reads were filtered to remove adaptor sequences and low-quality reads (>15% bases with a quality value <20 and >5% of the unknown sequences). Next, transcriptome assembly was completed by using the Trinity software. Subsequently, the GO, COG, KEGG, NR, Swiss-Prot, and Pfam databases were selected for the unigenes annotation.

### 4.2. Differential Expression Analysis and Validation of P450s

Since our prior work indicated P450s were involved in NTSR to mesosulfuron-methyl in *B. syzigachne*, P450s were selected as key enzymes for this study [20]. Unigene expression comparison was made between mesosulfuron-methyl treatment and CK (named as TR vs CK). The read count for each gene in each sample was obtained from the mapping results by RSEM (RNA-Seq by expectation maximization) [42]; then, the mapped read counts for each transcript were normalized, to eliminate the effects of the sequencing depth and gene length on the gene expression levels, by RPKM (reads per kilobase million mapped reads) [43]. Those contigs with a |log2 (fold change)| ≥ 1 and *p* value < 0.05 were identified as differentially expressed genes (DEGs) between TR and CK in this study.

To verify the expression patterns and accuracy of the experiment, we raised the screening standard for log2fold change over 2.50 DEGs for further verification. To test the expression levels of these genes, a new experiment was carried out. Parallel plant materials were replanted for the seed germination, plant cultivation, herbicide treatment, and total RNA extraction. Samples were taken after 12, 24 and 48 h of mesosulfuron-methyl treatment. About 29 P450s genes were selected for quantification by the qRT-PCR following previous methods [44]. The 18S rRNA was used as internal control genes in qRT-PCR [45]. For all genes, Oligo 7.0 was used to design qPCR primers (Supplementary Table S7).

### 4.3. Arabidopsis Transformation with P450 Genes in B. syzigachne

Of the differentially expressed P450 genes, *CYP704A177* and *CYP99A44* were selected for further functional verification based on gene expression values and sequence availability. Due to the complexity of the sequence, the complete coding sequence of the other P450 genes such as *CYP96B84*, *CYP71D7*, and *CYP93A1* were not obtained in this study. The coding region of *CYP99A44* and *CYP704A177* were amplified via PCR with the primer pairs of *CYP99A44* F/R (F: AATGGATCCATGGAGCTAAACACAGCTACCCTG; R: GCTCTAGATTAACTTTCCATGGGAACATTATATGG) and *CYP704A177* F/R (F: AATGGATCCATGGATTCACCGCTGAGC; R: GCTCTAGATTACGCACTTGATCGCCG) using cDNA prepared from two-three stage shoots. The primer pairs were flanking restriction site for the enzymes BamHI (TaKaRa, Catalog No. 1605) (forward underlined) and XbaI (TaKaRa, Catalog No. 1634) (reverse underlined). The amplified fragment was cloned into the pEASY^®^-Blunt Simple Cloning Vector (TransGen Biotech, Beijing, China), and transformed to *Escherichia coli* (DH5α, Shanghai Weidi Biotechnology Co., Ltd., Shanghai, China) to analyzed the sequences. Phylogenetic analysis was conducted using MEGA-X and iTOL (http://itol.embl.de (accessed on 9 October 2022)).

CYP-Blunt plasmid (CYP refers to the cytochrome P450 gene) samples were extracted using plasmid extraction kit (Vazyme Biotech Co., Ltd., Nanjing, China). The extracted plasmid and overexpression vector pPZP211-3Flag were double digested with BamHI and XbaI, and ligated using the T4 DNA Ligase Kit (TransGen Biotech, Beijing, China). Ligation products were introduced into DH5α cells and selected on a Luria-Bertani (LB) medium containing spectinomycin (60 μg/mL). The conformed plasmid pPZP211-35S::CYP was introduced into *Agrobacterium tumefaciens* strain GV3101 using the heat shock method, and transformed GV3101 was selected from a plate containing LB medium supplemented with spectinomycin (60 μg/mL) and rifampicin (20 μg/mL). The transformed GV3101 were introduced into *Arabidopsis* by the modified floral-dip method [46,47]. Plants were kept in dark for 48 h after being watered. Then, they were transferred in a growth chamber with conditions of steady 23 °C during the day or night, 50% relative humidity, and 16 h of light condition with 200 μmol m^−2^·s^−1^. Transgenic plants T1 containing kanamycin marker were selected with the plate containing MS medium supplemented with kanamycin (50 μg/mL). Leaf discs were collected from survivors for DNA extraction. DNA was extracted using the Plant Genomic DNA kit (Tiangen Biotech, Beijing, China) according to the manufacturer’s instructions. The extracted DNA was used as a template for PCR detection with the *CYP99A44* and *CYP704A177* F/R primers listed previously, and plants containing the foreign gene were transplanted and maintained. This process was repeated to obtain non-segregated T3 transgenic lines. Four to five 2-week seedlings of T3 homozygous lines grown on MS solid medium were harvested, frozen in liquid nitrogen, and stored for RNA extraction. cDNAs was synthesized as previously described [48]. *CYP99A44* and *CYP704A177* were amplified using PCR to confirm their successful transcription in *Arabidopsis*.

### 4.4. Resistance Identification of Transgenic Plants

WT *Arabidopsis* (columbia-0) and T3 seeds of pPZP211-35S::CYP *Arabidopsis* were surface-sterilized for 20 min in a solution of 4% sodium hypochlorite and washed with sterilized water. Susceptibility of transgenics to mesosulfuron-methyl was evaluated by growth on MS solid medium containing mesosulfuron-methyl at concentrations of 3.5 and 35 nM. The dishes in the light incubator were re-arranged every other day. At 14 d, the susceptibility of the transgenic *Arabidopsis* to mesosulfuron-methyl was visually assessed.

The susceptibility of transgenic *Arabidopsis* to mesosulfuron-methyl and other ALS inhibitors were determined by spraying whole plants when *Arabidopsis* was 30 d old [49]. Concentrations of mesosulfuron-methyl were 0.25, 0.5, 1.0, 2.0, and 4.0 g a.i./ha. Pyroxsulam, bispyribac-sodium, imazethapyr, and flucarbazone were tested at single doses of 2.81, 7.50, 18.75, and 7.875 g a.i./ha, respectively. At 14 d after treatment, the *Arabidopsis* shoots above ground level were cut, and their weights were measured and expressed as a percentage of untreated controls. Each harvest contained four replications and experiment was conducted twice. Because the interaction of herbicide treatment and experiment was not significant (*p* > 0.05), the data were pooled. The GR_50_ (the herbicide rate causing a 50% growth reduction in plants) was calculated by fitting the pooled data to a four-parameter log-logistic curve using SigmaPlot v.14.0 (Systat Software, San Jose, CA, USA) [50].

### 4.5. Subcellular Localization of CYP99A44 and CYP704A177

The full CDS (coding sequence) except for the stop codon of *CYP99A44* and *CYP704A177* were cloned into a pEGOEP vector to produce a fusion gene with GFP under the control of CaMV35S. The plasmids pEGOEP-35S: CYP-GFP were sequenced and concentrated for *Arabidopsis* protoplasts transformation; pEGOEP-35S: GFP was used as a background control and pEGOEP-35S: mCherry-mRFP was used as an endoplasmic reticulum marker, and protoplasts preparation was performed according to Yoo et al. [51]. The plasmids pEGOEP-35S: CYP-GFP and pEGOEP-35S: mCherry-mRFP or pEGOEP-35S: GFP alone were mixed with 120 μL 40% PEG-4000 and used for transformation with 16 h incubation in the dark. The fluorescence of the protoplasts was observed using confocal microscopy.

## 5. Conclusions

Overall, this study revealed the major gene contributing to the metabolic resistance of *B. syzigachne* to mesosulfuron-methyl. *CYP99A44* conferred transgenic *Arabidopsis* resistance to mesosulfuron-methyl, but not to pyroxsulam, imazethapyr, flucarbazone, or bispyribac-sodium. *CYP704A177* showed resistance to pyroxsulam and bispyribac-sodium but not to mesosulfuron-methyl as well as other ALS herbicides. Sequence analyses showed that *CYP99A44* and *CYP704A177* clustered in the CYP71 and CYP74 clan respectively, and both are located in the endoplasmic reticulum. To the best of our knowledge, this is the first report to identify resistance genes function of *B. syzigachne* to mesosulfuron-methyl and other ALS inhibitors. However, how gene catalyzes mesosulfuron-methyl metabolism needs further research.

## Figures and Tables

**Table 1:** Selection and validation of P450 genes in resistant *Beckmannia syzigachne* population when comparing those with and without herbicide treatment.

Gene ID	Function Annotation	RNA-Seq		Fold Change: qRT-PCR		
		Log2Fold Change	*p* Value	12 h	24 h	48 h
TRINITY_DN11725_c0_g1	*CYP71C4*	9.32	7.83 × 10^−22^	1.02	0.1	71.01 *
TRINITY_DN12889_c1_g2	*CYP99A44*	7.98	2.49 × 10^−14^	1.45	1.53	764.04 *
TRINITY_DN13855_c0_g2	*CYP72A15*	7.53	3.05 × 10^−18^	1.11	1.64	15.3 *
TRINITY_DN4168_c0_g1	*CYP71P11*	6.52	1.95 × 10^−24^	4.08 *	0.38	405.91 *
TRINITY_DN12796_c0_g1	*CYP71D55*	6.45	1.71 × 10^−19^	2.17 *	0.2	72.38 *
TRINITY_DN14232_c0_g3	*CYP71A1*	6.19	1.59 × 10^−30^	1.34	2.61 *	1.96
TRINITY_DN13855_c0_g1	*CYP72A14*	5.85	1.37 × 10^−22^	0.18	0.44	42.49 *
TRINITY_DN12677_c3_g5	*CYP71D55*	5.76	1.80 × 10^−12^	0.78	1.21	2.33 *
TRINITY_DN11507_c1_g9	*CYP93A1*	5.63	1.49 × 10^−07^	2.04 *	6.93 *	376.11 *
TRINITY_DN13492_c1_g1	*CYP71D7*	5.51	4.32 × 10^−11^	8.38 *	9.4 *	596.34 *
TRINITY_DN11999_c1_g1	*CYP71Z7*	5.22	8.45 × 10^−15^	0.44	1.93	1.22
TRINITY_DN12352_c4_g3	*CYP89E28*	5.1	4.31 × 10^−07^	2.11 *	0.87	86.37*
TRINITY_DN13661_c0_g3	*CYP72A14*	5.05	3.19 × 10^−33^	1.44	0.77	1.34
TRINITY_DN11183_c0_g1	*CYP96B84*	4.79	3.08 × 10^−24^	9.63 *	66.26 *	4837.35 *
TRINITY_DN13666_c3_g5	*CYP94A1*	4.51	2.53 × 10^−09^	0.99	1.21	1.36
TRINITY_DN11248_c6_g1	*CYP74A4*	4.39	7.35 × 10^−19^	1.21	1.38	2.21 *
TRINITY_DN13901_c6_g2	*CYP93A1*	4.22	7.27 × 10^−34^	1.55	1.68	2.01 *
TRINITY_DN13830_c0_g10	*CYP72A219*	3.97	3.11 × 10^−63^	1.65	4.62 *	13.43 *
TRINITY_DN12172_c1_g2	*CYP734A6*	3.78	1.64 × 10^−15^	2.22 *	1.25	1.64
TRINITY_DN12282_c2_g1	*CYP72A603*	3.09	3.29 × 10^−13^	1.09	1.76	26.13 *
TRINITY_DN8749_c0_g3	*CYP71A9*	3.09	9.20 × 10^−06^	1.14	0.26	20.61 *
TRINITY_DN13317_c1_g3	*CYP71A1*	3.07	1.07 × 10^−11^	0.57	0.17	4.74 *
TRINITY_DN12014_c1_g2	*CYP71Z7*	2.97	1.78 × 10^−38^	1.43	2.24 *	1.79
TRINITY_DN9718_c0_g3	*CYP76B6*	2.86	2.18 × 10^-4^	1.21	1.65	1.77
TRINITY_DN11725_c0_g2	*CYP71C4*	2.74	4.73 × 10^−07^	0.66	1.56	1.92
TRINITY_DN13955_c0_g1	*CYP99A2*	2.61	1.56 × 10^-2^	0.36	0.79	1.65
TRINITY_DN8888_c0_g1	*CYP716B2*	2.61	4.15 × 10^-3^	1.8	0.23	77.84 *
TRINITY_DN12495_c1_g4	*CYP704A177*	2.57	4.65 × 10^−29^	5.29 *	4.45 *	7.99 *
TRINITY_DN12889_c0_g6	*CYP71D55*	2.54	3.09 × 10^−05^	0.55	0.68	1.23

*p* value of <0.05 is indicated by * from the SPSS analysis.

**Table 2:** Herbicide sensitivity of wild type and transgenic *Arabidopsis* expressing *CYP99A44* and *CYP704A177* to mesosulfuron-methyl.

Arabidopsis		Mesosulfuron-Methyl	
	GR_50_ ± SE (g a.i./ha) ^1^	GR_90_ ± SE (g a.i./ha) ^2^	Resistance Index ^3^
WT	1.28 ± 0.15	8.99 ± 4.98	1.00
CYP99A44	2.88 ± 0.34	13.17 ± 3.46	2.25
CYP704A177	1.83 ± 0.76	5.85 ± 1.54	1.43

^1^ GR_50_, herbicide rate causing 50% growth reduction of plants; ^2^ GR_90_, herbicide rate causing 90% growth reduction of plants; ^3^ Resistance index, was calculated by dividing GR_50_ value of the transgenic *Arabidopsis* by that of the WT.

## Data Availability

Not applicable.

## Supplementary Materials

The following supporting information can be downloaded at: https://www.mdpi.com/article/10.3390/ijms232012175/s1.
