# Supplementary material for: Cytochrome P450 BsCYP99A44 and BsCYP704A177 Confer Metabolic Resistance to ALS Herbicides in Beckmannia syzigachne
Source: Int J Mol Sci. 2022 Oct 12;23(20):12175. doi: 10.3390/ijms232012175 (PMC9602494; doi:10.3390/ijms232012175)

Supplementary Table S1. Summary of transciptome sequencing data and transcriptome assembly of resistant *Beckmannia syzigachne*.

Sample	Clean reads	Clean bases	Error rate (%)	Q20 (%)	Q30 (%)	GC Content (%)
CK1	29779024	4468806566	0.0249	98.05	94.21	45.23
CK2	29781410	4465891195	0.0252	97.93	93.95	45.4
CK3	29801578	4457081328	0.0246	98.18	94.57	45.42
TR1	29672594	4453976162	0.0257	97.7	93.46	45.18
TR2	29788322	4457464497	0.0251	97.75	94.09	45.63
TR3	29637080	4429808607	0.0261	97.56	93.15	45.56

Note: CK, untreated *B. syzigachne*; TR, treated *B. syzigachne* with mesosulfuron-methyl.

Supplementary Table S2. The data table of RNA-seq from resistant *Beckmannia syzigachne*.

Type	Unigene
Total unigene number	53651
Total unigene base	51772083
Largest	15352
Smallest	201
Average length	964.98
N50	1670
E90N50	2219
GC percent (%)	41.46
TransRate score	0.30006
BUSCO score	82.2% (2.8%)

Supplementary Table S3. Up-expressed P450s genes in resistant *Beckmannia syzigachne* population when comparing with and without herbicide treatment

Unigene ID	Gene annotation	Log_2Fold Change	P value
TRINITY_DN11725_c0_g1	P450 71C4	9.32	$7.83E^{-22}$
TRINITY_DN12889_c1_g2	P450 99A2	7.98	$2.49E^{-14}$
TRINITY_DN13855_c0_g2	P450 72A15	7.53	$3.05E^{-18}$
TRINITY_DN4168_c0_g1	P450 71A9	6.52	$1.95E^{-24}$
TRINITY_DN12796_c0_g1	P450 71D55	6.45	$1.71E^{-19}$
TRINITY_DN13618_c0_g4	P450 71C4	6.45	$3.84E^{-18}$
TRINITY_DN14232_c0_g3	P450 71A1	6.19	$1.59E^{-30}$
TRINITY_DN13855_c0_g1	P450 72A14	5.85	$1.37E^{-22}$
TRINITY_DN12677_c3_g5	P450 71D55	5.76	$1.80E^{-12}$
TRINITY_DN11507_c1_g9	P450 93A1	5.63	$1.49E^{-07}$
TRINITY_DN13492_c1_g1	P450 71D7	5.51	$4.32E^{-11}$
TRINITY_DN11999_c1_g1	P450 71Z7	5.22	$8.45E^{-15}$
TRINITY_DN12352_c4_g3	P450 89A2	5.10	$4.31E^{-07}$
TRINITY_DN13661_c0_g3	P450 72A14	5.05	$3.19E^{-33}$
TRINITY_DN11183_c0_g1	P450 96A15	4.79	$3.08E^{-24}$

TRINITY_DN13666_c3_g5	P450 94A1	4.51	$2.53E^{-09}$
TRINITY_DN11248_c6_g1	P450 74A4	4.39	$7.35E^{-19}$
TRINITY_DN13901_c6_g2	P450 93A1	4.22	$7.27E^{-34}$
TRINITY_DN13830_c0_g10	P450 72A15	3.97	$3.11E^{-63}$
TRINITY_DN12172_c1_g2	P450 734A6	3.78	$1.64E^{-15}$
TRINITY_DN12282_c2_g1	P450 72A15	3.09	$3.29E^{-13}$
TRINITY_DN8749_c0_g3	P450 71A9	3.09	$9.20E^{-06}$
TRINITY_DN13317_c1_g3	P450 71A1	3.07	$1.07E^{-11}$
TRINITY_DN12014_c1_g2	P450 71Z7	2.97	$1.78E^{-38}$
TRINITY_DN9718_c0_g3	P450 76B6	2.86	$2.18E^{-04}$
TRINITY_DN11725_c0_g2	P450 71C4	2.74	$4.73E^{-07}$
TRINITY_DN8888_c0_g1	P450 716B2	2.61	$4.15E^{-03}$
TRINITY_DN12495_c1_g4	P450 704C1	2.57	$4.65E^{-29}$
TRINITY_DN12889_c0_g6	P450 71D55	2.54	$3.09E^{-05}$
TRINITY_DN11725_c0_g4	P450 71C2	2.48	$2.18E^{-04}$
TRINITY_DN13618_c0_g8	P450 71D7	2.46	$2.65E^{-05}$
TRINITY_DN13765_c0_g2	P450 74A2	2.39	$4.99E^{-09}$
TRINITY_DN14160_c2_g1	P450 72A219	2.20	$2.81E^{-11}$
TRINITY_DN8634_c0_g1	P450 704C1	2.14	$5.54E^{-24}$
TRINITY_DN10765_c0_g3	P450 72A63	1.94	$1.04E^{-08}$
TRINITY_DN12172_c1_g10	P450 709B2	1.90	$5.12E^{-05}$
TRINITY_DN13765_c0_g1	P450 74A2	1.71	$2.38E^{-06}$
TRINITY_DN13666_c3_g6	P450 86A2	1.54	$7.46E^{-03}$
TRINITY_DN13806_c1_g5	P450 89A2	1.53	$6.56E^{-04}$
TRINITY_DN13793_c1_g1	P450 71A24	1.47	$4.36E^{-04}$
TRINITY_DN12236_c1_g1	P450 81E1	1.46	$5.31E^{-04}$
TRINITY_DN9697_c0_g1	P450 90B1	1.35	$4.92E^{-06}$
TRINITY_DN10753_c1_g4	P450 94B3	1.27	$8.17E^{-03}$
TRINITY_DN13317_c1_g6	P450 71A1	1.26	$3.47E^{-03}$
TRINITY_DN11507_c1_g7	P450 78A9	1.24	$2.67E^{-05}$
TRINITY_DN8888_c0_g2	P450 716B2	1.17	$5.37E^{-03}$
TRINITY_DN5662_c0_g1	P450 711A1	1.10	$5.38E^{-05}$

Supplementary Table S4. Up-expressed GST genes in resistant *Beckmannia syzigachne* population when comparing with and without herbicide treatment

Unigene ID	Gene annotation	Log_2Fold Change	P value
TRINITY_DN10876_c6_g1	GSTU1	8.22	$2.88E^{-16}$
TRINITY_DN10463_c0_g5	GSTU6	8.13	$1.27E^{-47}$
TRINITY_DN4076_c0_g1	GSTU17	6.34	$6.16E^{-31}$
TRINITY_DN10463_c0_g8	GSTU6	5.97	$1.22E^{-29}$
TRINITY_DN12523_c0_g7	GSTU6	5.92	$1.01E^{-58}$
TRINITY_DN13151_c2_g11	GSTU6	5.63	$1.09E^{-25}$
TRINITY_DN13151_c2_g5	GSTU6	5.55	$1.69E^{-27}$
TRINITY_DN13455_c2_g1	GST 23	5.40	$5.50E^{-13}$

TRINITY_DN13202_c1_g1	GSTF2	4.98	$2.65E^{-07}$
TRINITY_DN12523_c0_g14	GSTU6	4.95	$8.37E^{-07}$
TRINITY_DN13202_c2_g1	GSTF1	4.88	$1.47E^{-13}$
TRINITY_DN10463_c0_g14	GSTU6	4.61	$2.79E^{-83}$
TRINITY_DN472_c0_g1	GSTF2	4.46	$2.56E^{-20}$
TRINITY_DN11954_c0_g2	GSTF2	4.38	$2.58E^{-38}$
TRINITY_DN13276_c0_g1	GSTU6	4.17	$9.53E^{-26}$
TRINITY_DN10463_c0_g4	GSTU6	3.99	$1.45E^{-19}$
TRINITY_DN10831_c0_g8	GSTF1	3.70	$4.28E^{-04}$
TRINITY_DN10463_c0_g19	GST 23	3.20	$1.54E^{-14}$
TRINITY_DN10876_c4_g1	GSTU6	3.18	$1.55E^{-03}$
TRINITY_DN10463_c0_g2	GSTU6	3.11	$5.93E^{-04}$
TRINITY_DN13202_c0_g1	GSTF2	2.98	$1.23E^{-08}$
TRINITY_DN10831_c0_g5	GSTU6	2.71	$2.13E^{-10}$
TRINITY_DN10831_c0_g4	GSTU6	2.61	$1.80E^{-09}$
TRINITY_DN12214_c1_g1	GSTBZ2	2.10	$6.73E^{-03}$
TRINITY_DN10831_c0_g6	GSTU6	1.72	$5.98E^{-09}$
TRINITY_DN12820_c1_g1	GSTU6	1.56	$1.34E^{-11}$
TRINITY_DN13052_c1_g4	GSTU6	1.37	$3.96E^{-04}$
TRINITY_DN10747_c0_g11	GSTU6	1.30	$4.52E^{-11}$

Supplementary Table S5. Up-expressed GTs genes in resistant *Beckmannia syzigachne* population when comparing with and without herbicide treatment

Unigene ID	Gene annotation	Log_2Fold Change	P value
TRINITY_DN10220_c0_g4	GTs 18	1.76	0.0006626
TRINITY_DN10262_c0_g2	GTs 64	1.361	$1.79E^{-10}$
TRINITY_DN10352_c1_g1	GTs 4	2.556	$4.59E^{-14}$

Supplementary Table S6. Up-expressed ABC transporter genes in resistant *Beckmannia syzigachne* population when comparing with and without herbicide treatment

Unigene ID (ABC transporter name)	SwissProt	Log_2Fold Change	P value
TRINITY_DN13035_c0_g1	ABCB4	6.07	$5.42E^{-08}$
TRINITY_DN13035_c0_g5	ABCB4	5.47	$3.07E^{-64}$
TRINITY_DN13192_c0_g1	ABCE2	4.98	$2.79E^{-24}$
TRINITY_DN10735_c1_g3	ABCC9	4.12	$9.13E^{-45}$
TRINITY_DN13035_c0_g2	ABCB4	2.71	$1.53E^{-07}$
TRINITY_DN13756_c1_g2	ABCB11	2.60	$6.67E^{-14}$
TRINITY_DN9158_c0_g3	ABCF4	2.47	$8.93E^{-16}$
TRINITY_DN11770_c3_g7	ABCF4	1.89	$6.41E^{-16}$
TRINITY_DN11641_c0_g3	ABCF1	1.75	$4.07E^{-10}$
TRINITY_DN13356_c0_g1	ABCF1	1.28	$6.33E^{-06}$
TRINITY_DN11641_c0_g5	ABCF1	1.11	$3.09E^{-14}$

Supplementary Table S7. Primers used for RT-qPCR analysis of the P450s genes in *Beckmannia syzigachne* Steud.

Gene name	Direction	Sequence (5'-3')
18S rRNA	Forward	AGAAACGGCTACCACATC
	Reverse	CCAAGTCCAACTACGAG
CYP704A177	Forward	AGGCTCCTCAACATCTCGTC
	Reverse	GGCGATCACGAAATTCAGTA
CYP716B2	Forward	TCAAGGAGAAGCGTGCAAAG
	Reverse	GTGCTGGATGAGGAAGGTGA
CYP71A1	Forward	AGTATCTCAAGGCGGTGCTG
	Reverse	CGTTGATGAAGAGCGTGGTC
CYP71A9	Forward	CTGGTGCTTGCCAACTTGCT
	Reverse	GGCCAGCAGCACTAGAGGGT
CYP72A603	Forward	AGGCAGGAGCAACTAACGAA
	Reverse	CCAATTCTGGGTCAGGGATC
CYP72A219	Forward	CAAGGGAGGAGGTACTATGC
	Reverse	CAAATATCAGGGTCATGGTG
CYP96B84	Forward	AACCTGCGACCCAATGAATA
	Reverse	TGGCATACAGGTCGAACACG
CYP99A44	Forward	ATTCCCCAGTGGCAACACAA
	Reverse	GACATGATCCACTCGACGCT
CYP89E28	Forward	CTCACGGACGCCGAGATGGT
	Reverse	CAGCACGACGGCCTTGAGGT
CYP71P11	Forward	GCGAGTGCTTCCTCAACCTG
	Reverse	GTGCTTTCCGCTGATGTGCT
CYP72A14	Forward	TGACCATCACCGATCCTAAC
	Reverse	ATTGAGTCCATCCGCTAACA
CYP93A1	Forward	GCTTCGCCTTCGCACCATAC
	Reverse	CTCCTCCGTCACGCTTCCTG
CYP71C4	Forward	GAGCGGCATGGCCTACCTCA
	Reverse	CGAACGGCACGAACTGGAAA
CYP71D55	Forward	CCAAGGCACAGCAAGAAGTT
	Reverse	TAGCCAAGCGGATGAATAGA
CYP71D7	Forward	CGCTACTGGCCCTTTGTTTC
	Reverse	CGTGGGTCTTCAGCATCTCC

Supplementary Figure S1. Volcano plots depicting differential gene expression between CK and TR group of *Beckmannia syzigachne*. The x-axis shows the log2 fold change or relative abundance. The P value (-log base 10) for differential gene expression is plotted on the y-axis.

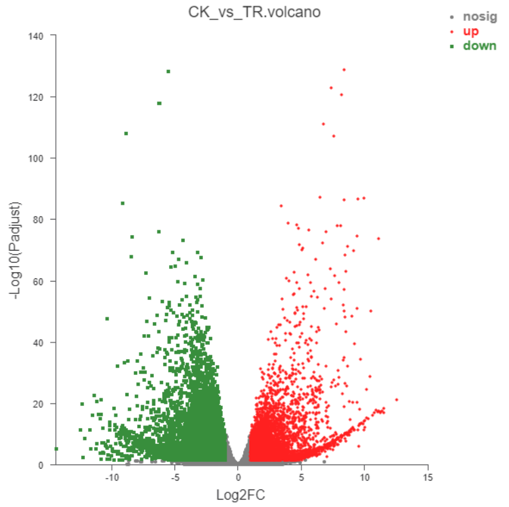

Supplementary Figure S2. Conserved domains analysis of *CYP99A44* (A) and *CYP704A177* (B) in *Beckmannia syzigachne*.

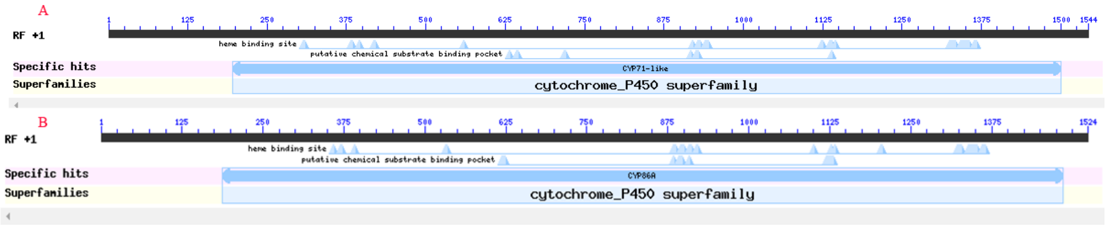

Supplementary Figure S3. Subcellular localization of *CYP704A177*. A: pEGOEP-35S: CYP704A177-GFP shown in green; B: endoplasmic reticulum (ER) marker mCherry-mRFP shown in red; C: Chloroplast auto-fluorescence; D: the bright-field; E: merged images; F: pEGOEP-35S: GFP shown in green; G: endoplasmic reticulum (ER) marker mCherry-mRFP shown in red; H: the bright-field; I: merged images. Scale bars: 10 µm in A-I.

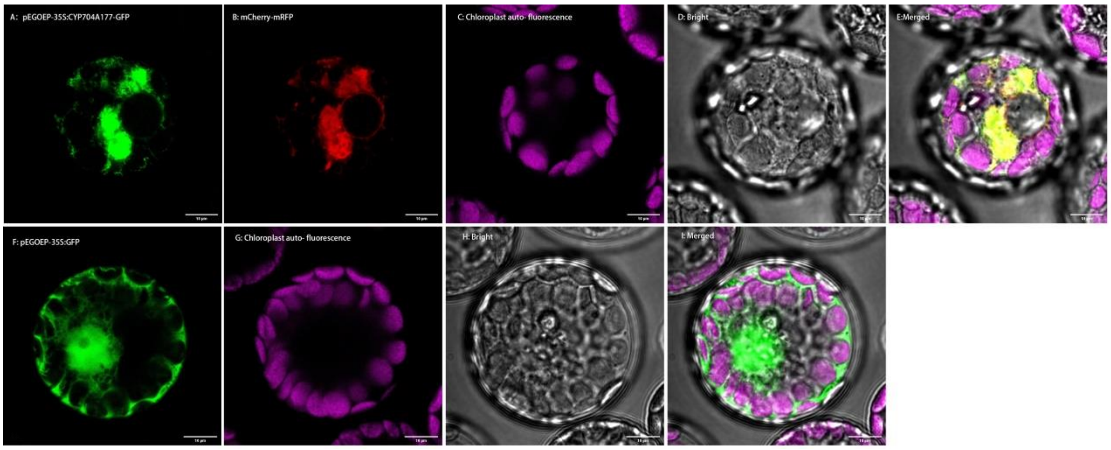

Supplement: Supplementary file 1 [file ijms-23-12175-s001.zip › ijms-1908184-supplementary.pdf]
